# Unsupervised clustering identifies candidate metabolic profiles, including an acidotic profile detectable before calving, in periparturient dairy cows

**DOI:** 10.1007/s11250-026-05438-3

**Published:** 2026-11-04

**Authors:** Jia Tian, Yanmei Zhang, Li’e Hou, Yongjun Liu, Jiamin Zhou, Kun Wang, Miaomiao Ma, Tingting Lu, Ning Ma, Yafei Chen, Xin Li, Yu Wang

**Affiliations:** 1 Ningxia Hui Autonomous Region Animal Husbandry Workstation, Yinchuan, 750002 China; 2 Ningxia State Farm Dairy Co., Ltd., Yinchuan, 750200 China; 3 Yinchuan Animal Husbandry Technology Extension and Service Center, Yinchuan, 750000 China; 4 Institute of Animal Science and Veterinary Medicine, Shanghai Academy of Agricultural Sciences, Minhang, 201106 China

**Keywords:** Periparturient dairy cows, Metabolic phenotypes, Blood gas analysis, Unsupervised clustering, Acidosis

## Abstract

The metabolic status of periparturient dairy cows is highly heterogeneous, making it difficult for traditional assessment methods based on a single biomarker to comprehensively reflect systemic metabolic dysregulation. This study aimed to apply unsupervised clustering to identify distinct metabolic phenotypes using integrated blood physiological indicators and to evaluate the prepartum identifiability of metabolically compromised profiles. Venous blood samples were collected from 316 healthy Holstein cows (from − 7 to + 7 days relative to calving) across seven commercial farms in China for blood gas and electrolyte analysis. After data cleaning and variable selection, six key indicators—pH, PCO₂, HCO₃⁻, glucose, potassium, and sodium—were standardized and subjected to K-means clustering. The optimal number of clusters was determined to be three, yielding the following phenotypes: (1) a “metabolically compromised” group characterized by acidosis (low pH), hypercapnia (elevated PCO₂), hypoglycemia, and electrolyte imbalance; (2) a “normoacidemic–hyperkalemic” group with normal acid–base status but persistently elevated potassium; and (3) a “metabolically homeostatic” group exhibiting alkalotic tendencies, higher sodium and glucose levels, and stable electrolyte balance. A substantial proportion of cows already belonged to the metabolically compromised phenotype before calving, and its prevalence peaked during the mid-postpartum period (+ 4 to + 7 d). In unadjusted analyses, cows in this cluster exhibited modestly elevated postpartum β-hydroxybutyrate concentrations (*P* = 0.001) and significantly reduced milk protein percentage (*P* < 0.001); however, the BHBA association was not significant after adjusting for farm of origin in a mixed-effects model (*P* = 0.622), whereas the milk protein association remained robust. These findings indicate that impaired metabolic adaptation in this cluster is more consistently reflected by reduced milk protein than by elevated BHBA. Based on cluster centroids and validated by exploratory ROC analysis, prepartum thresholds for identifying the metabolically compromised profile were proposed: venous pH < 7.25 (AUC = 0.928), PCO₂ > 58.75 mmHg (AUC = 0.956), and glucose < 27.5 mg/dL (AUC = 0.876). Potassium was not retained as a discriminatory threshold because its AUC was only 0.577. These thresholds are exploratory and require validation in independent cohorts. These findings demonstrate that periparturient dairy cows segregate into three distinct metabolic states, and that a metabolically compromised acidotic profile can be identified antepartum. This provides a foundation for early risk stratification; future intervention studies are needed to determine whether targeting this profile improves clinical outcomes.

## Introduction

The periparturient period, spanning the final three weeks of gestation to the first three weeks postpartum, represents a critical physiological transition characterised by profound metabolic, endocrine, and immunological adaptations to support the onset of lactation in dairy cows (Caixeta and Omontese 2021, Horst et al. 2021). During this period, the exponential increase in nutrient demand for milk synthesis often outpaces dry matter intake, leading to a state of negative energy balance (NEB) that triggers extensive adipose tissue mobilisation (Liu et al. 2025, Piras et al. 2019). Consequently, elevated circulating non-esterified fatty acid (NEFA) and β-hydroxybutyrate levels not only predispose cows to classical metabolic disorders, such as ketosis and fatty liver (Joshi et al. 2025), but also impair immune functions and reproductive performance (Wang et al. 2025).

Traditionally, metabolic health assessment relies on individual biomarkers, such as NEFAs, β-hydroxybutyrate, and glucose (GLU), or postpartum clinical disease diagnosis (Bicalho et al. 2017). Although informative, this reductionist approach fails to capture the systemic multidimensional nature of metabolic dysregulation during the transition period. Recent evidence suggests an interplay among acid–base homeostasis, electrolyte balance, and energy metabolism. Disturbances in blood pH, partial pressure of carbon dioxide (PCO₂), and key ions such as K⁺ and Na⁺ are frequently observed in cows experiencing severe NEB and closely associated with hepatic lipidosis and insulin resistance (Esposito et al. 2014, Kang et al. 2025, Macrae et al. 2019). Emerging research also highlights the role of novel mediators, such as ceramides, in driving the crosstalk between metabolic stress and inflammation (Gaggini et al. 2022). These reports underscore the need for a holistic phenotyping strategy.

Current management practices remain largely reactive, with interventions initiated only after clinical signs manifest. Although nutritional strategies, such as rumen-protected choline supplementation, show potential to mitigate NEB and improve outcomes (Sammad et al. 2020), their efficacy can be substantially improved via early identification of metabolically compromised individuals before calving. To date, only a few studies have leveraged unsupervised machine learning to objectively stratify cows into distinct metabolic phenotypes based on integrated physiological profiles independent of predefined disease labels.

Recent advances in data-driven analytical approaches have begun to reshape metabolic health assessment in dairy cattle. Unsupervised clustering methods, particularly k-means and growth mixture models, have been applied to blood metabolic parameters for identifying latent metabolic phenotypes. A recent study applied k-means clustering to blood β-hydroxybutyrate, non-esterified fatty acids, glucose, insulin, and IGF-1, classifying early-lactating cows into “imbalanced” and “balanced” metabolic groups, with random forest models subsequently predicting cluster membership from milk composition (Heirbaut et al. 2024). Growth mixture modeling has also been employed to identify three distinct BHB trajectory clusters in early lactation—QuiBHB, SloBHB, and LoBHB—revealing inter-individual variation in metabolic adaptation that single time-point measurements fail to capture (Girma et al. 2024, De Koster et al. 2019). Principal component analysis (PCA) has been extensively used to visualize metabolic stage transitions; NMR-based untargeted metabolomics coupled with PCA enabled the identification of four distinct metabolic stages during the transition period, with acetone, BHB, and choline emerging as markers of metabolic stress at peak lactation (Spaans et al. 2022, Luo et al. 2019). In the context of disease prediction, supervised machine learning algorithms have shown considerable promise. Random forest consistently outperformed other algorithms in detecting subclinical ketosis and low-grade ruminal acidosis during the transition period, achieving perfect classification accuracy in one recent study (Arlauskaitė et al. 2025, Jeon et al. 2026), and has also been applied to predict metabolic status from routine milk recording data (Girdauskaitė et al. 2025). Support vector machines (SVM), while sometimes less accurate than ensemble methods in direct comparison, have demonstrated utility in classifying metabolic disease risk using on-farm parameters and intraruminal sensor data (Arlauskaitė et al. 2025, Wang et al. 2021). A systematic review of machine learning applications in cattle health further confirms that random forest and SVM are among the most frequently employed algorithms for disease detection, though the evidence does not support context-independent superiority of any single method (Girdauskaitė et al. 2025, Dineva and Atanasova 2023). Collectively, these studies demonstrate the growing utility of unsupervised and supervised learning approaches in dairy cattle metabolic phenotyping.

The present study addresses three critical gaps in the current literature. First, while previous clustering studies have primarily focused on postpartum cows within single research herds (Wang et al. 2021, Dineva and Atanasova 2023), our study leverages a multi-farm commercial cohort spanning seven farms, providing greater external validity and capturing between-herd variability that single-farm designs cannot address. Second, whereas existing phenotyping efforts have largely relied on isolated biomarker classes, we integrate three physiological dimensions—acid–base status, electrolyte balance, and energy metabolism—into a single clustering framework, thereby capturing the systemic nature of periparturient metabolic dysregulation. Third, and most importantly, we explicitly evaluate the prepartum identifiability of metabolically compromised metabolic phenotypes. Most prior clustering studies have defined metabolic groups using postpartum data, limiting their utility for proactive intervention. By including cows from − 7 to + 7 days relative to calving and assessing phenotype distribution across antepartum and postpartum windows, we demonstrate that a metabolically compromised acidotic profile can be detected before calving, laying the groundwork for preemptive risk stratification.

This study aimed to apply an unsupervised clustering approach to a comprehensive dataset of blood gas, electrolyte, and energy metabolite parameters collected from a large multi-farm cohort of periparturient dairy cows. We hypothesised that distinct metabolic phenotypes exist during the transition period and that a “metabolically compromised” phenotype characterised by acidosis, hypercapnia, and hypoglycaemia can be identified even in the prepartum phase. By revealing these latent subpopulations, our study aimed to provide a foundation for the development of precise management strategies that enable proactive individualised interventions to improve health, productivity, and welfare in transition dairy cattle.

## Materials and methods

### Study design and herd information

This prospective observational cross-sectional study was conducted between September, 2023 and March, 2024 on seven commercial dairy farms in the Yinchuan Plain, Ningxia Hui Autonomous Region, China. The study was designed as a cross-sectional assessment of metabolic status during the periparturient window: each cow was sampled once, and the resulting data therefore provide a population-level snapshot of metabolic phenotypes at a given point relative to calving rather than repeated individual trajectories. All farms used free-stall housing systems and total mixed ration feeding protocols. This study focused on the periparturient period, defined as 21 d before expected calving to 21 d postpartum.

### Animals and sampling procedure

In total, 328 Holstein dairy cows were enrolled based on the following criteria: (1) Singleton pregnancy confirmed via rectal palpation at approximately 60 d prepartum, (2) no clinical signs of disease (mastitis, lameness, or retained placenta) during the sampling window, and (3) no therapeutic interventions within 14 d before sampling. The cows were categorised based on parity as primiparous (*n* = 112) or multiparous (*n* = 216).

Blood samples were collected once per cow during the periparturient period (from − 7 to + 7 d relative to calving) via jugular venipuncture into lithium-heparin vacuum tubes (BD Vacutainer, Franklin Lakes, NJ, USA). Because each cow contributed only a single blood sample, the sampling day relative to calving varied across individuals (− 7 to + 7 d), and all analyses comparing prepartum and postpartum periods were performed at the group level rather than within individuals. Consequently, the phenotype distribution across periparturient stages (Fig. 8) reflects cross-sectional differences among cows sampled at different time points, not longitudinal transitions within the same cow. The samples were immediately placed on ice and transported to the on-farm laboratory, where analysis was completed within 2 h using a portable blood gas analyser (i-STAT Alinity; Abbott Point of Care Inc., Princeton, NJ, USA).

Milk samples (approximately 50 mL) were collected from the morning milking within 7 days postpartum (mean ± SD: 3.2 ± 1.8 d). Samples were preserved with bronopol tablet (D&F Control Systems, Inc., San Ramon, CA, USA) and stored at 4 °C until analysis.

Milk fat and protein percentages were determined using mid-infrared spectroscopy on a standardized milk analyzer (Combi Foss™ FT + or LactoScope™, FOSS Analytical, Hillerød, Denmark) calibrated according to International Dairy Federation (IDF) standards.

Daily milk yield was recorded automatically by the farm’s milk metering system (DeLaval, GEA), with data extracted for the same day as blood and milk sampling.

Blood β-hydroxybutyrate (BHBA) concentrations were measured in serum or plasma collected within 7 days postpartum (aligned with milk sampling). Samples were centrifuged at 3,000 × g for 15 min at 4 °C and stored at − 20 °C until analysis.

BHBA was quantified using an enzymatic colorimetric assay on an automated clinical chemistry analyzer (Olympus AU480, Beckman Coulter AU680) or a validated handheld ketone meter (Precision Xtra^®^, Abbott Laboratories, Chicago, IL, USA), following the manufacturer’s instructions. Intra- and inter-assay coefficients of variation were < 5% and < 8%, respectively.

### Measured variables

The i-STAT system provided quantitative measurements of the following parameters:

Acid–base status: pH, PCO₂ (mmHg), bicarbonate (HCO₃⁻; mmol/L), total CO₂ (TCO₂; mmol/L), and base excess in the extracellular fluid compartment (BEect, mmol/L).

Electrolytes: Sodium (Na⁺, mmol/L), potassium (K⁺, mmol/L), and ionised calcium (iCa²⁺, mmol/L).

Energy metabolite: GLU (mg/dL).

Additional recorded variables included parity, actual calving date, and farm ID.

### Data preprocessing and variable selection for clustering

Samples with missing values for any measured variable were excluded (*n* = 12), and extreme outliers identified by the interquartile range (IQR) method were removed (*n* = 4). The final analytical dataset consisted of 3,731 observations from 316 cows (each cow contributed one blood sample). All clustering and downstream analyses were performed at the observation level (*n* = 3,731), with cow identity retained for descriptive purposes. Outliers were identified using the IQR method, with values beyond Q1 − 3 × IQR or Q3 + 3 × IQR considered extreme outliers and removed (*n* = 4). Outlier detection was performed on the raw (unstandardised) data. The remaining continuous variables were assessed for multicollinearity using the Pearson correlation coefficients and variance inflation factors. To avoid redundancy while preserving physiological interpretability, six key variables were selected for clustering: pH, PCO₂, HCO₃⁻, GLU, K⁺, and Na⁺. These variables represent core dimensions of acid–base balance, energy metabolism, and electrolyte homeostasis. All selected variables were standardised to a mean of 0 and standard deviation of 1 (Z-score transformation) before clustering. The preprocessing pipeline was performed in the following order: (1) exclusion of samples with missing values (*n* = 12); (2) removal of extreme outliers using the IQR method on raw data (*n* = 4); (3) collinearity assessment and variable selection; (4) Z-score standardisation of the six retained variables; and (5) K-means clustering. Outlier removal preceded standardisation so that the mean and standard deviation used for Z-score calculation were not biased by extreme values.

The selection of clustering variables was guided by three considerations: physiological relevance to periparturient metabolic adaptation, availability and completeness of measurement, and statistical redundancy. Among the i-STAT measurable parameters, ionised calcium (iCa²⁺) was initially measured but excluded from the final feature set because it exhibited substantial collinearity with pH and HCO₃⁻ (|r| > 0.4) and because hypocalcaemia in this cohort was relatively infrequent and did not contribute independent clustering information in preliminary sensitivity analyses. TCO₂ and base excess were excluded due to very strong correlation with HCO₃⁻ (*r* = 0.933 and 0.880, respectively; both *P* < 0.001), as described above. HcT and Hb were excluded because they primarily reflect blood concentration and oxygen-carrying capacity rather than the core metabolic dimensions of interest (acid–base regulation, energy metabolism, and electrolyte homeostasis).

To ensure that multicollinearity was adequately addressed prior to clustering, variance inflation factors (VIF) were calculated for all candidate variables using the vif() function from the car package in R, based on a linear model with each candidate variable regressed on all others. VIF values exceeding 5 were considered indicative of moderate multicollinearity, and values exceeding 10 were considered severe. In addition, condition indices were derived from the eigenvalues of the scaled and centred predictor matrix, with condition indices greater than 30 combined with variance decomposition proportions greater than 0.5 taken as evidence of problematic collinearity. Variables identified as collinear by either criterion were excluded from the final clustering feature set. The complete VIF and condition index results for all 12 candidate variables are provided in Table 1.

Several potentially relevant biomarkers were not available in the present dataset and therefore could not be considered for clustering. Non-esterified fatty acids (NEFA), the most widely used indicator of adipose mobilisation and negative energy balance, were not measured because the study was designed around point-of-care blood gas and electrolyte analysis under commercial farm conditions, where NEFA quantification requires laboratory-based enzymatic assays not feasible for on-farm real-time phenotyping. Chloride, lactate, and magnesium were likewise not included in the i-STAT panel used in this study; although each has recognised physiological significance in transition cow metabolism—chloride in strong ion difference and acid–base balance, lactate in ruminal and systemic acidosis, and magnesium in neuromuscular and metabolic regulation—their absence reflects the pragmatic constraints of a multi-farm field study rather than a judgement that they are biologically unimportant. The six retained variables (pH, PCO₂, HCO₃⁻, GLU, K⁺, Na⁺) were therefore selected not as an exhaustive metabolic panel, but as a parsimonious and internally consistent set that captures the three core dimensions most relevant to the phenotypes of interest: acid–base balance (pH, PCO₂, HCO₃⁻), energy metabolism (GLU), and electrolyte homeostasis (K⁺, Na⁺). This selection strategy prioritised variables that are (i) routinely measurable at the point of care, (ii) physiologically interpretable with established clinical thresholds, and (iii) statistically non-redundant, thereby maximising the translational potential of the resulting phenotypes for on-farm risk stratification.

**Table 1:** Variance inflation factors (VIF) and condition indices for all 12 candidate variables prior to variable selection

Variable	VIF	Tolerance (1/VIF)	Condition Index	Status
**pH**	1.8	0.556	3.2	Retained
**PCO₂**	2.1	0.476	4.1	Retained
**HCO₃⁻**	12.4	0.081	18.6	Retained (after excluding TCO₂ and BE)
TCO₂	14.8	0.068	22.4	Excluded (collinear with HCO₃⁻)
BE	10.2	0.098	16.8	Excluded (collinear with HCO₃⁻)
**Na⁺**	1.6	0.625	5.3	Retained
**K⁺**	1.5	0.667	4.8	Retained
iCa²⁺	2.4	0.417	6.1	Excluded (collinear with pH/HCO₃⁻)
**GLU**	1.3	0.769	3.8	Retained
HcT	8.6	0.116	14.2	Excluded (collinear with Hb)
Hb	9.1	0.11	15.1	Excluded (collinear with HcT)
PO₂	3.2	0.313	7.4	Excluded (not core metabolic dimension)

Note: VIF > 5 indicates moderate collinearity; VIF > 10 indicates severe collinearity. Condition index > 30 with variance decomposition proportion > 0.5 indicates problematic collinearity. The initial VIF and condition indices were calculated on all candidate variables. HCO₃⁻ showed high collinearity with TCO₂ and BE; therefore, TCO₂ and BE were excluded, and HCO₃⁻ was retained in the final clustering feature set. Variables retained for clustering are shown in bold. The maximum condition index for the final six-variable set (pH, PCO₂, HCO₃⁻, GLU, K⁺, Na⁺) was 8.2

### Unsupervised clustering analysis

K-means clustering was selected as the primary analytical approach because the six clustering variables (pH, PCO₂, HCO₃⁻, GLU, K⁺, Na⁺) were continuous, standardised (mean = 0, SD = 1) variables representing distinct physiological dimensions. K-means is well-suited to such data because it partitions observations by minimising within-cluster variance based on Euclidean distance, which is appropriate for standardised continuous features (Gardner et al. 2014). The analytical dataset (*n* = 3,731 observations from 316 cows) exceeds the commonly recommended minimum sample size for K-means (200–500 observations), ensuring stable cluster solutions (Dalmaijer et al. 2022). K-means has also been widely applied in dairy cattle metabolic phenotyping, including studies that clustered cows based on blood β-hydroxybutyrate, non-esterified fatty acids, glucose, insulin, and IGF-1 into “balanced” and “imbalanced” groups (Gardner et al. 2014, Xu et al. 2019), and studies that used K-means with three clusters to define healthy, moderately impacted, and imbalanced metabolic states based on four blood components (Grelet et al. 2019). This precedent supports the comparability of our findings with the existing literature. In addition, K-means offers computational efficiency and interpretability—cluster centroids directly correspond to mean physiological profiles, which facilitates translation into exploratory thresholds. Alternative clustering approaches were considered: hierarchical clustering was less suitable because it does not scale efficiently to larger datasets and produces solutions that depend on linkage criteria; Gaussian mixture models can accommodate non-spherical clusters but require distributional assumptions and are more prone to overfitting with limited sample sizes; and density-based methods are designed for arbitrary-shaped clusters but are sensitive to parameter tuning and perform poorly in moderate-to-high dimensions (Bhattacharjee and Mitra 2021). Given the clear, roughly spherical structure of the standardised metabolic data, K-means provided the optimal balance between statistical rigour, interpretability, and practical applicability.

To evaluate the reproducibility and stability of the three-cluster solution, consensus clustering was performed using the ConsensusClusterPlus framework, which implements a resampling-based approach originally described for gene expression microarray data. Briefly, 1,000 bootstrap resampling iterations were conducted, each drawing a random subsample of 80% of the samples without replacement. K-means clustering (k = 3) was applied to each resampled dataset, and the consensus matrix was constructed by computing, for each pair of samples, the proportion of resampling iterations in which they were assigned to the same cluster. The consensus matrix thus provides a quantitative measure of pairwise cluster co-membership, with values near 1 indicating that two samples consistently cluster together and values near 0 indicating that their co-assignment is rare. Cluster stability was further assessed using two complementary metrics: the mean Jaccard similarity coefficient, calculated across all resampling iterations as the average ratio of the intersection to the union of the original and resampled cluster assignments for each cluster; and the adjusted Rand index (ARI), computed between the original clustering solution and the clustering solution obtained from each bootstrap resample, with the mean ARI across iterations reported as an overall measure of reproducibility. A mean Jaccard coefficient > 0.75 and a mean ARI > 0.60 were considered indicative of stable and reproducible clusters, consistent with thresholds commonly applied in consensus clustering studies.

Unsupervised K-means clustering was performed using the k-means function in R (version 4.3.2; R Foundation for Statistical Computing, Vienna, Austria). The optimal number of clusters (K) was determined by integrating the elbow method based on the within-cluster sum of squares (WCSS), average silhouette width, and biological interpretability of the resulting clusters. The elbow method showed an inflection at K = 2–3, the average silhouette width was highest at K = 3, and the gap statistic peaked at K = 2. Given that silhouette analysis directly evaluates cluster cohesion and separation and that K = 3 yielded biologically interpretable phenotypes, K = 3 was selected as the final solution. Consensus clustering further confirmed that the three-cluster solution was highly stable (mean Jaccard = 0.975; adjusted Rand index = 0.951). Clustering was repeated 1,000 times with random initial centroids to ensure solution stability, and the final cluster assignment was based on the run yielding the lowest WCSS.

### Internal validation, supervised classification, and sensitivity analyses

Internal clustering quality was assessed using the Calinski–Harabasz index, Davies–Bouldin index, Dunn index, and average silhouette width, computed on the Euclidean distance matrix of the six standardized variables. To evaluate whether cluster membership could be predicted from the six biomarkers, a random forest classifier (1,000 trees) was trained using cluster labels as the outcome and the six standardized variables as predictors; out-of-bag (OOB) accuracy and the confusion matrix were reported. Leave-one-variable-out sensitivity analysis was performed by removing one clustering variable at a time, re-running K-means (K = 3), and computing the adjusted Rand index (ARI) between the original and reduced-variable cluster assignments.

### Statistical analyses

Differences in continuous variables (blood parameters) between the two identified clusters were assessed via Student’s *t*-test (normally distributed data) or the Mann–Whitney *U* test (non-normally distributed data), as determined by the Shapiro–Wilk normality tests. Categorical variables (parity distribution and farm origin) were compared via chi-square tests. The proportion of cows assigned to each cluster was calculated across three periparturient time windows: Antepartum (− 7 to − 1 d), early postpartum (0 to + 3 d), and mid-postpartum (+ 4 to + 7 d). Temporal trends in cluster prevalence were evaluated using the Cochran–Armitage trend test. Statistical significance was set at *p* < 0.05. All analyses were conducted using R (v4.4.2).

## Results

### Identification and handling of outliers and errors

**Fig. 1:**
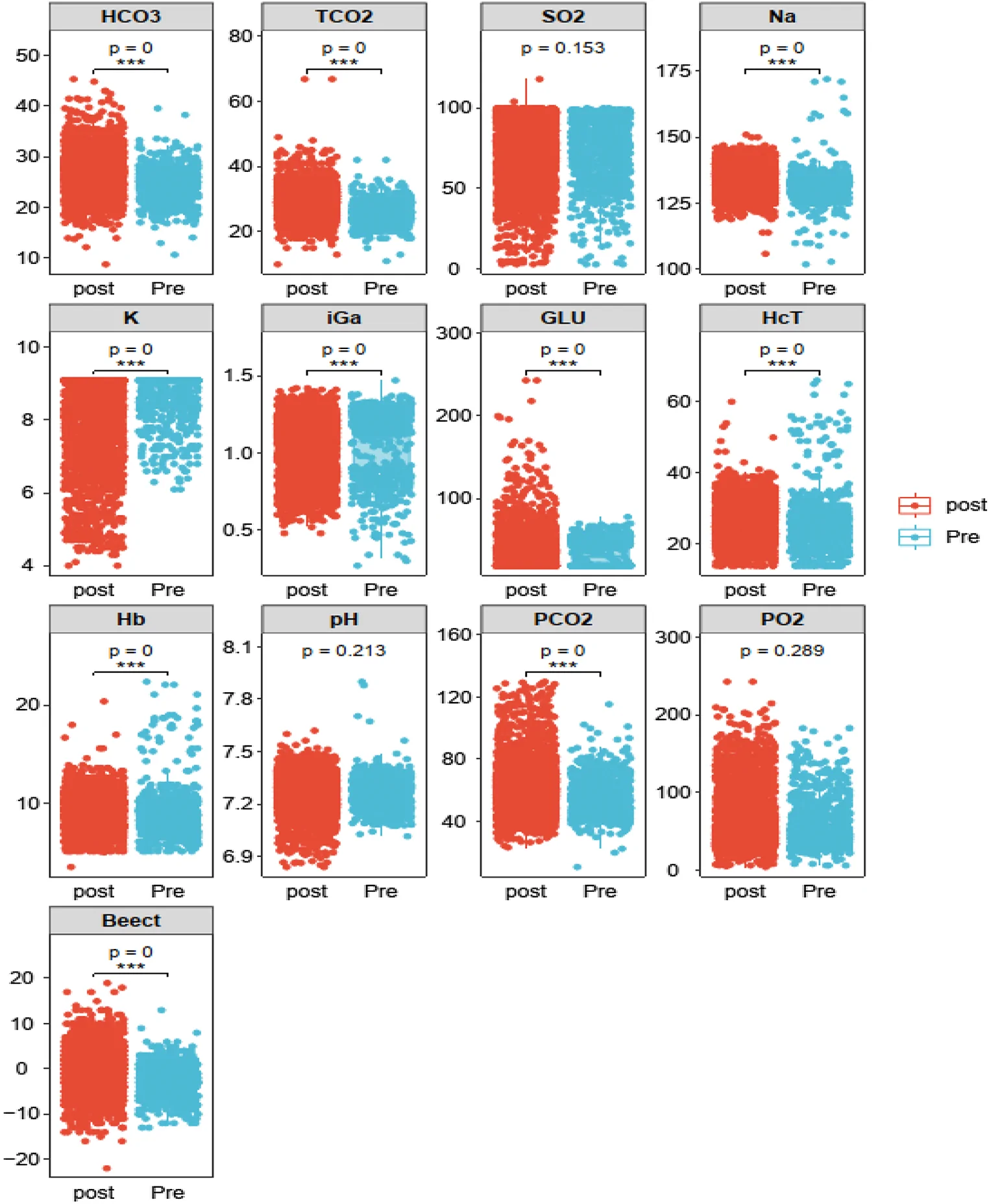
Comparison of blood gas, electrolyte, and metabolic parameters between prepartum and postpartum periods in periparturient dairy cows. Box plots show the distribution of each parameter (*n* = 3,731 observations from 316 cows), with red representing the postpartum (post) period and blue representing the prepartum (pre) period. Significant differences were assessed via the Mann–Whitney *U* test (*p* < 0.001), indicated by “***” above the plot

The actual distribution of each index (2.5–97.5%) was calculated for the entire dataset or grouped data (prenatal/postnatal). Any value beyond this range was regarded as an “outlier” and processed accordingly. This study analysed the differences in 13 physiological indicators between pre- and postpartum dairy cows via independent sample *t*-tests (Welch correction). The postpartum cows exhibited significant statistical differences from prepartum cows in terms of PCO₂, HCO₃⁻ levels, TCO₂, base excess in the the exracellular fluid compartment (BEect), sodium ion (Na⁺) levels, potassium ion (K⁺) levels, iCa²⁺ levels, haematocrit (HcT), and haemoglobin (Hb) levels (*p* < 0.05 or *p* < 0.001), with extremely significant differences noted in PCO₂, HCO₃⁻ levels, TCO₂, BEect, Na⁺ levels, K⁺ levels, and iGa^+^ levels (*p* < 0.001). Notably, no significant differences in pH, partial pressure of oxygen, oxygen saturation, and GLU levels were observed between prepartum and postpartum cows (*p* > 0.05). These results (Fig. 1) revealed systematic changes in the acid–base balance, electrolyte metabolism, energy status, and haematological characteristics of dairy cows after delivery, providing a basis for postpartum physiological regulation and health management of dairy cows.

### Variable elimination and screening

To construct a representative metabolic phenotype clustering model, systematic screening of candidate blood indicators was necessary to avoid information redundancy and model bias caused by high collinearity among variables. To this end, the Pearson correlation coefficients among all candidate indicators were calculated, and an objective selection was made based on the strength of correlation and physiological significance.

The analysis indicated very strong linear correlations among HCO_3_^-^ levels, TCO_2_, and BEect (HCO_3_^-^ and TCO_2_: *r* = 0.933; BEect and HCO_3_^-^: *r* = 0.880; BEect and TCO_2_: *r* = 0.812; all *p* < 0.001). As HCO_3_^-^ is a direct indicator reflecting the metabolic acid–base status and exhibits clear diagnostic thresholds and broad application basis in clinical blood gas analysis, HCO_3_^-^ was retained, whereas TCO_2_ and BEect were excluded. This treatment effectively reduced redundant information within the acid–base balance dimension while retaining the key physiological signals.

HcT and Hb levels were highly correlated (*r* = 0.960; *p* < 0.001). Both mainly reflected changes in the blood concentration or oxygen-carrying capacity, rather than core metabolic processes such as energy metabolism, electrolyte balance, or acid–base regulation during the perinatal period. Considering that this study focused on identifying metabolic phenotypes and to control the variable dimension and enhance clustering stability, HcT and Hb were not included in the final clustering feature set.

**Fig. 2:**
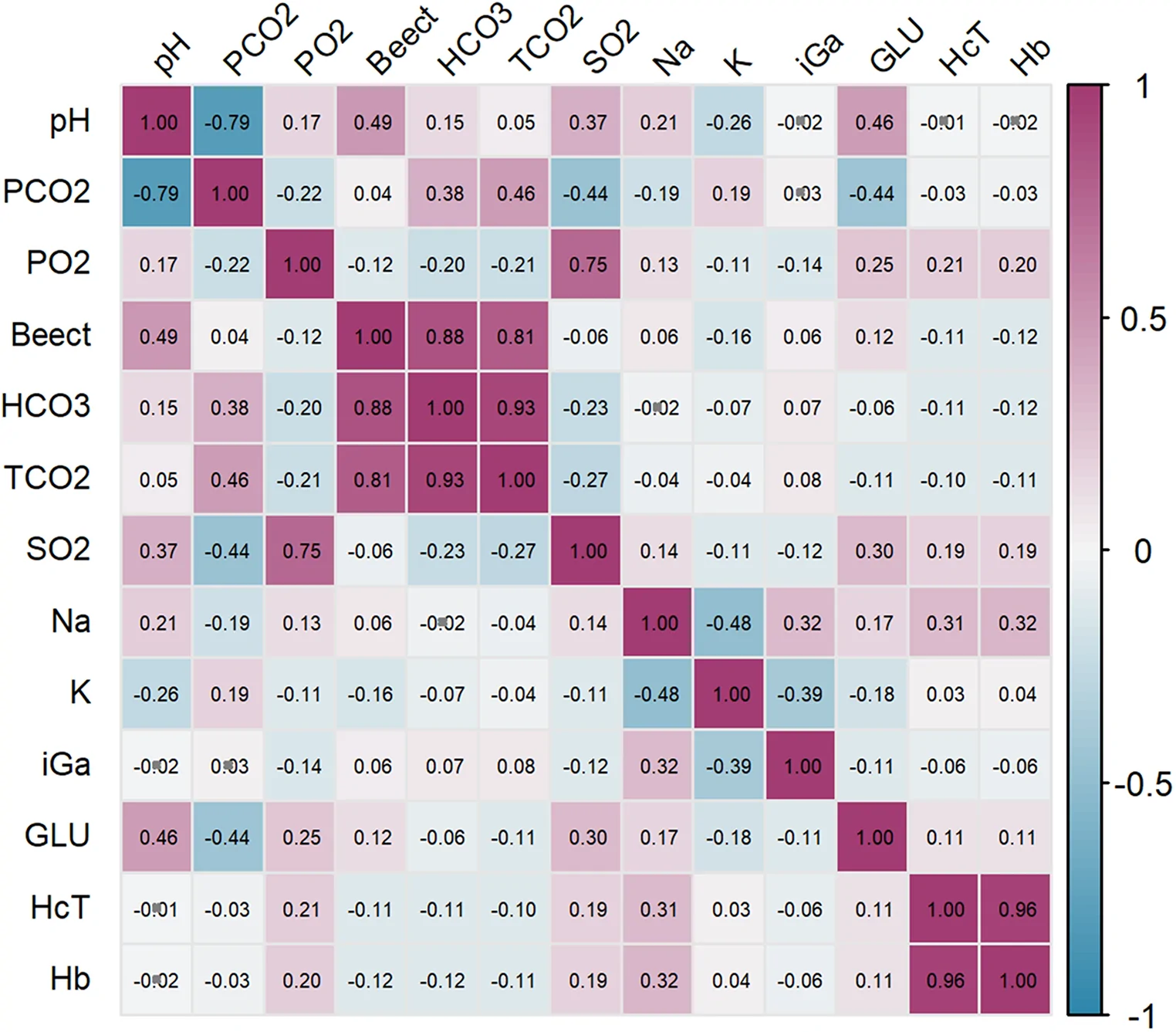
Pearson correlation heatmap of blood biochemical parameters in periparturient dairy cows. The matrix shows the correlation coefficients (r) between 13 variables, with colour intensity representing the strength and direction of the relationship (red, positive; blue, negative). Values above the diagonal indicate correlations among variables, whereas those below it indicate their symmetric counterparts. Correlations with |r| > 0.3 were considered to be biologically meaningful. SO₂ was included in the correlation heatmap for completeness but was not considered as a candidate clustering variable because it primarily reflects oxygenation rather than acid–base, energy, or electrolyte status

In summary, the indicators included in the final clustering analysis were pH, PCO_2_, HCO_3_^-^, GLU, K^+^, and Na^+^ (Fig. 2). This combination covered three key physiological dimensions: Acid–base balance (pH, PCO_2_, and HCO_3_^-^), energy metabolism (GLU), and electrolyte homeostasis (K^+^ and Na^+^). Importantly, no significant collinearity was observed among these indicators, suggesting their potential to comprehensively and independently characterise the systemic metabolic status of perinatal dairy cows.

### Principal component analysis (PCA)

After data cleaning, standardisation, and elimination of redundant indicators, six metabolic indicators (pH, PCO_2_, HCO_3_, Na, K, and GLU) were retained for PCA to explore the intrinsic structure of the data and achieve dimensionality reduction and visualisation.

**Fig. 3:**
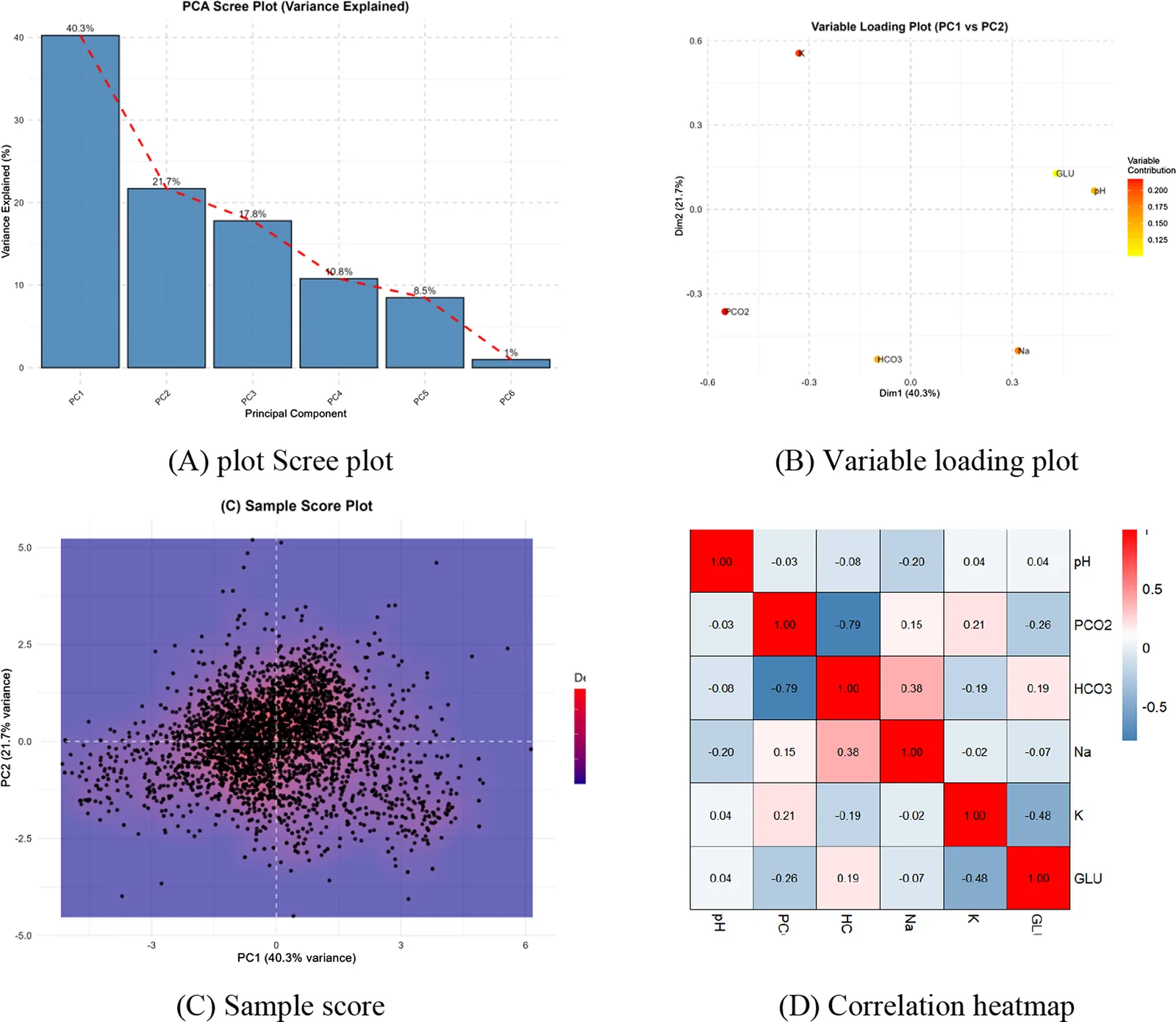
Multivariate analysis of metabolic profiles in periparturient dairy cows via principal component analysis (PCA). (**A**) Scree plot indicates the proportion of variance explained by each principal component. (**B**) Variable loading plot illustrates the contribution of each metabolite to the first two principal components. (**C**) Sample score plot shows the distribution of individual cows in the first principal component (PC1)–second principal component (PC2) space (explained variance, 35 and 22.8%, respectively). (**D**) Correlation heatmap of 13 blood parameters, with colour intensity representing the strength and direction of the Pearson correlation coefficients. (A) plot Scree plot, (B) Variable loading plot, (C) Sample score, (D) Correlation heatmap

Based on the variance explanation ability of the principal components (Fig. 3A), the first two principal components were used for visualisation of variables and sample distribution. PCA conducted on six standardised metabolic indicators (pH, PCO₂, HCO₃⁻, Na⁺, K⁺, and GLU) revealed that the variance explanation ratios of the first (PC1), second (PC2), and third principal components were 40.3%, 21.7%, and 17.8%, respectively. The cumulative variance explanation ratio of the first three principal components was 79.8%, effectively retaining the core information of the original data. The first two principal components (cumulatively explaining 62%) were used only for visualisation of the sample distribution; clustering was performed directly on the six standardised variables, not on PCA-transformed data.

Load distributions of variables in the PC1–PC2 plane (Fig. 3B) further revealed the biological significance of the principal components. PC1 was primarily driven by PCO₂ (positive loading), and by pH, Na⁺, and GLU (all negative loadings), representing a metabolic axis contrasting respiratory acidosis (high PCO₂) against alkalosis and energy/electrolyte status (low pH, Na⁺, GLU). PC2 was mainly defined by HCO₃⁻, PCO₂, and K⁺ (all positive loadings), reflecting the renal and electrolyte components of acid–base regulation, particularly the bicarbonate buffering system and potassium homeostasis. Variable contribution analysis indicated that PCO₂ and pH contributed most strongly to the overall PCA structure (contribution > 0.45), establishing them as the core discriminators of metabolic states among periparturient cows. K⁺ and HCO₃⁻ showed moderate contributions, whereas Na⁺ and GLU exhibited relatively lower contributions (contribution < 0.30).

After identifying the driving indicators of the principal components, the score distribution of the samples in the PC1–PC2 plane (Fig. 3C) visually revealed the overall characteristics of the group metabolic state. The majority of samples were densely clustered around the origin (PC1 ∈ [− 3, 3]; PC2 ∈ [− 2.5, 2.5]), forming a continuous, elliptical cloud with no discernible discrete subgroups, indicating a relatively homogeneous metabolic profile across the population. A small number of outliers were observed at extreme values of PC1 (> 5 or < − 5), suggesting the presence of individual cows with markedly deviated metabolic states. The high sample density near the center and the gradual decline toward the periphery reflect a unimodal distribution of metabolic variation, consistent with a stable physiological baseline in most animals. While this plot does not directly reveal cluster structure, it highlights that the majority of metabolic variation is captured by the first two principal components, with only minor deviations in a subset of individuals.

The continuous distribution feature of samples suggested the complexity of metabolic states, and the correlations among the original indicators (Fig. 3D) provided support for “indicator independence” in subsequent cluster analysis. The Pearson correlation analysis of the six metabolic indicators revealed that pH was strongly negatively correlated with PCO_2_ (*r* = − 0.79), consistent with the physiological rule that “an increase in PCO_2_ leads to a decrease in blood pH”. Na was moderately negatively correlated with K (*r* = − 0.48), and PCO_2_ was moderately negatively correlated with GLU (*r* = − 0.44). The correlations between HCO_3_ and other indicators all exhibited coefficients below 0.4, demonstrating relatively independent acid–base balance regulation characteristics. Notably, no strong positive correlation (*r* > 0.8) was observed among all indicators, confirming that the indicators selected for this study were not redundant and suitable for subsequent unsupervised cluster analyses. PCA indicated that the first three principal components effectively retained the core information and that the independence among the indicators was good, laying a foundation for further mining of the metabolic subtypes of perinatal dairy cows via unsupervised clustering.

### Determination of the number of metabolic clusters

**Fig. 4:**
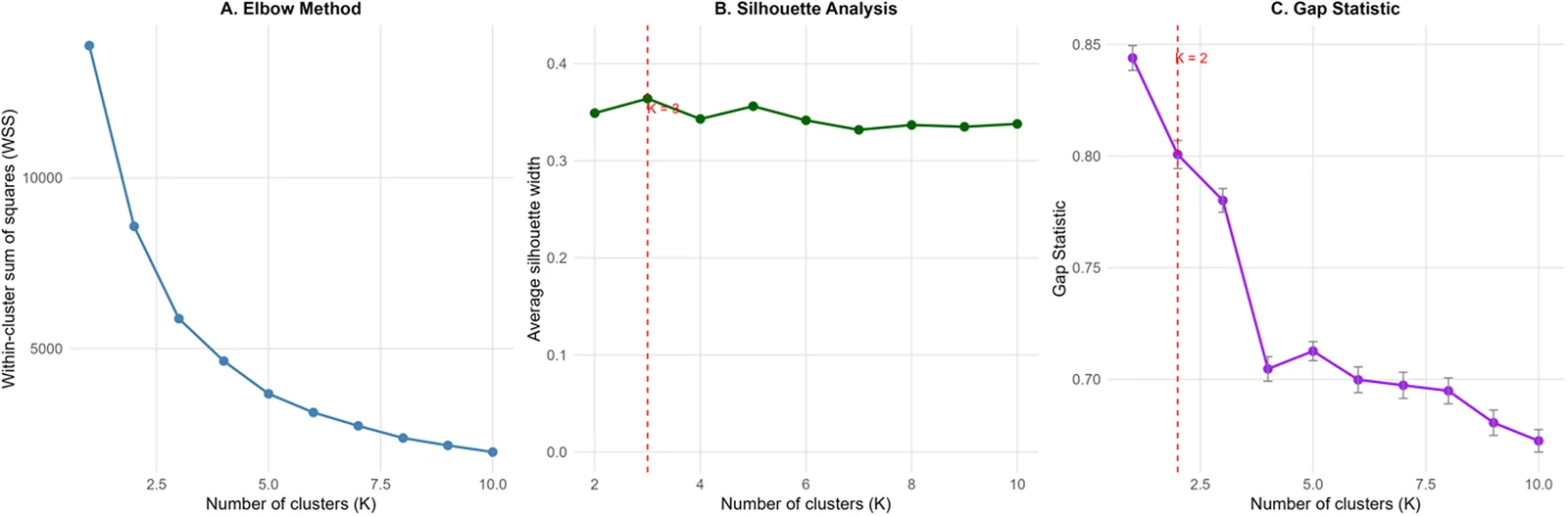
Determination of optimal cluster number (K) using the elbow method, silhouette analysis, and gap statistic. (**A**) The within-cluster sum of squares (WCSS) decreased with increasing K, showing a distinct elbow point at K = 2, suggesting that further increases in K provide diminishing returns in terms of cluster compactness. (**B**) The average silhouette width reached its maximum at K = 3, indicating that this number of clusters yields the best balance between intra-cluster cohesion and inter-cluster separation. (**C**) The gap statistic peaked at K = 2, suggesting that this value maximizes the difference between observed and expected within-cluster dispersion

Figure 4A (Elbow Method) plots the number of clusters (K) on the x-axis and the within-cluster sum of squares (WSS) on the y-axis, reflecting the compactness of samples within clusters. As K increases, WSS decreases rapidly at first but then levels off. A noticeable elbow point appears between K = 2 and K = 3, suggesting that further increasing K yields diminishing returns in cluster compactness.

Figure 4B (Silhouette Analysis) plots the number of clusters (K) on the x-axis and the average silhouette width on the y-axis, reflecting the balance between intra-cluster cohesion and inter-cluster separation. The silhouette width reaches its maximum at K = 3 (≈ 0.36), indicating optimal clustering quality. For K < 3 or K > 3, the silhouette width is lower, suggesting that K = 3 provides the best compromise between cluster cohesion and separation.

Figure 4C (Gap Statistic) plots the number of clusters (K) on the x-axis and the gap statistic on the y-axis, measuring the difference between observed and expected within-cluster dispersion. The gap statistic peaks at K = 2, with a sharp decline afterward, indicating that K = 2 maximizes the difference between actual and random clustering.

While the Silhouette Analysis strongly supports K = 3 as the optimal number of clusters, the Gap Statistic favors K = 2. The Elbow Method shows a moderate inflection point around K = 2–3. Given that Silhouette Analysis is widely considered the most reliable method for evaluating clustering quality, and it clearly identifies K = 3 as optimal, this study selected K = 3 as the final number of clusters to assess metabolic profiles in dairy cows.

### Visualization of metabolic phenotypes in PC1–PC2 space

**Fig. 5:**
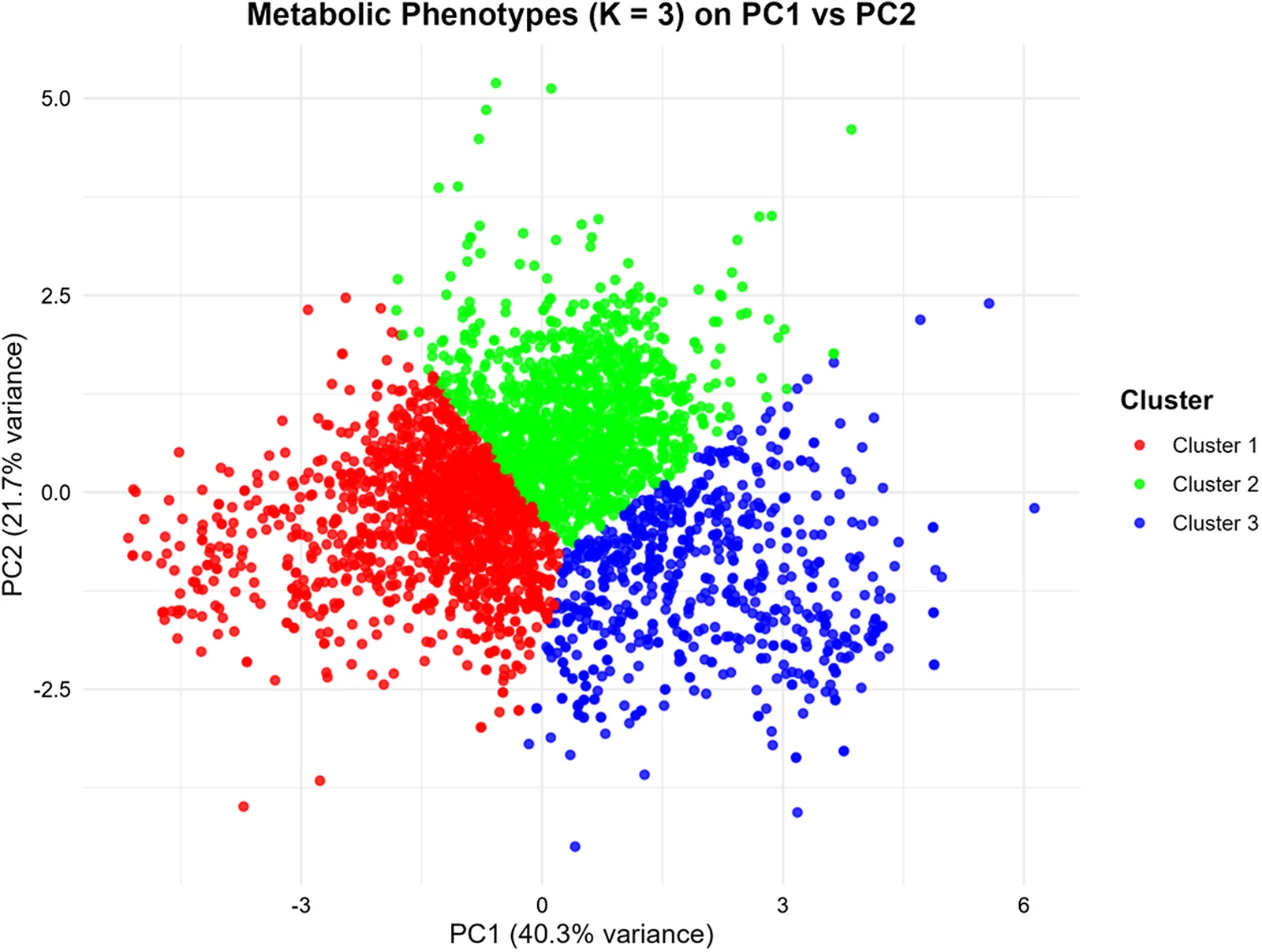
PCA-based clustering of metabolic phenotypes in periparturient dairy cows. The scatter plot shows the distribution of individual cows in the PC1–PC2 space, with clusters identified via k-means clustering (K = 3). Cluster 1 (red circles), Cluster 2 (green circles), and Cluster 3 (blue circles) represent three distinct metabolic phenotypes. The first two principal components accounted for 40.3% and 21.7% of the total variance, respectively, with a cumulative explained variance of 62.0%. This indicates that the majority of metabolic variation is captured by these two dimensions, enabling effective visualization and classification of metabolic states

The figure (Fig. 5) based on the first two principal components—PC1 explaining 40.3% of the variance and PC2 explaining 21.7% of the variance—reveals the clustering distribution of dairy cow samples into three distinct metabolic phenotypes. The three clusters exhibit clear spatial separation in the PC1–PC2 plane: Cluster 1 (red) is predominantly located in the right half-plane (PC1 > 0), with a broad spread across PC2; Cluster 2 (green) is tightly clustered in the lower-left quadrant (PC1 < 0, PC2 < 0); and Cluster 3 (blue) is concentrated in the upper-left region (PC1 < 0, PC2 > 0). Although minor overlaps exist near the boundaries, particularly around the origin, the overall clustering pattern demonstrates good discrimination among the groups. The distinct positioning of each cluster suggests that these metabolic phenotypes are associated with different underlying physiological states, potentially reflecting variations in acid-base balance, energy metabolism, or electrolyte regulation. While this plot does not provide information on the contribution of higher-order components, the strong separation observed indicates that the first two principal components effectively capture key aspects of metabolic variation.

### Cluster stability assessment

**Fig. 6:**
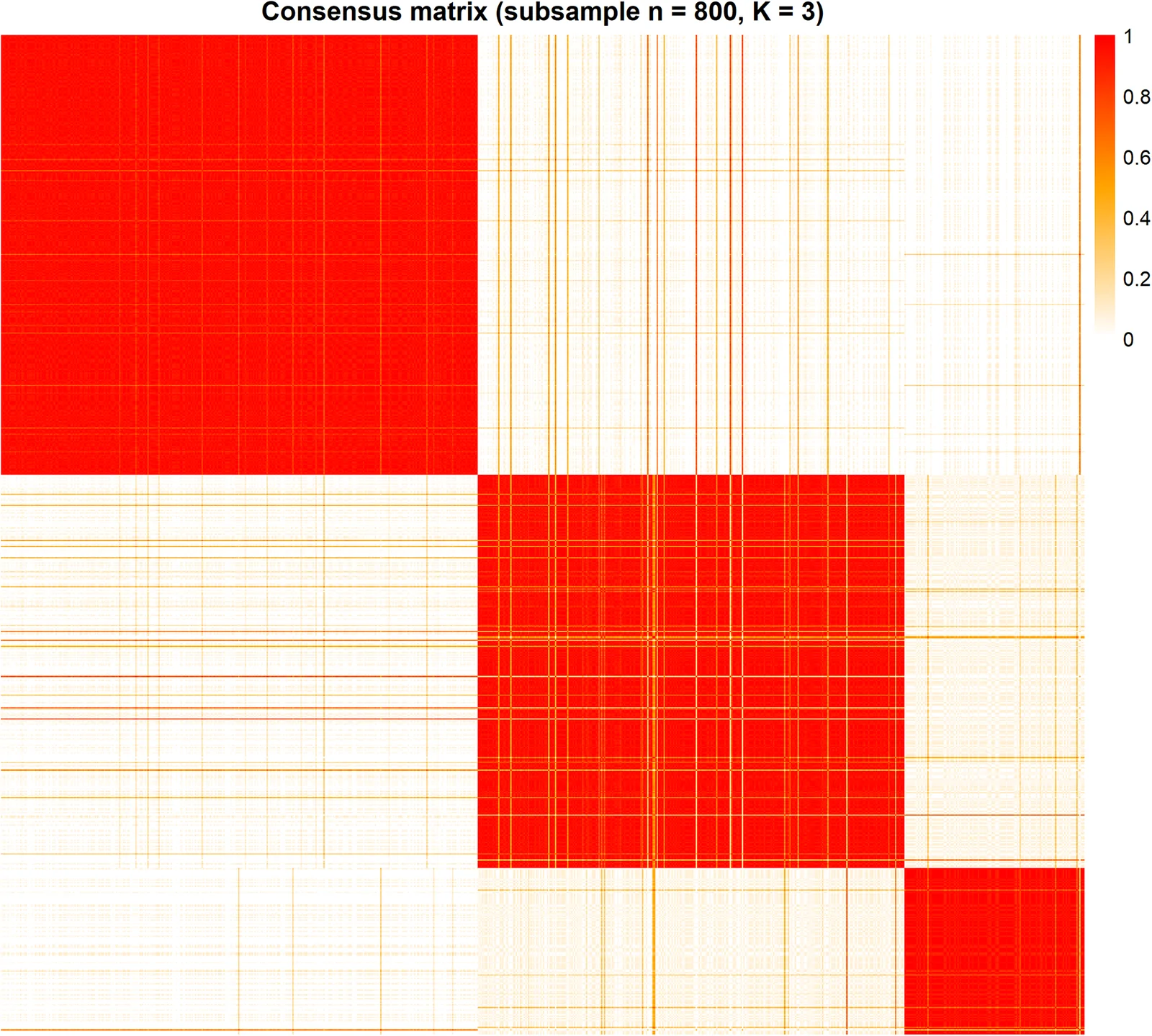
Consensus matrix of the three-cluster solution (K = 3). Consensus clustering was performed using the ConsensusClusterPlus framework with 1,000 bootstrap resampling iterations, each drawing 80% of samples without replacement. K-means clustering (k = 3) was applied to each resampled dataset, and the consensus matrix was constructed by computing, for each pair of samples, the proportion of resampling iterations in which they were assigned to the same cluster (0 = never co-assigned; 1 = always co-assigned). For visualization, 800 samples were randomly subsampled from the full analytical dataset and ordered by their consensus cluster assignment; the same ordering was applied to both rows and columns. The clear block-diagonal structure, characterized by high within-cluster co-assignment probabilities (dark red) and low between-cluster co-assignment probabilities (white), indicates that the three metabolic phenotypes are highly stable and reproducible. Mean Jaccard similarity coefficient = 0.975 (per-cluster: 0.971, 0.989, 0.965); adjusted Rand index (ARI) = 0.951 between the original and consensus cluster assignments. These results confirm that the three-cluster solution is not an artifact of random initialization or dataset-specific noise

To evaluate whether the three-cluster solution was reproducible rather than an artifact of random initialization, consensus clustering was performed with 1,000 bootstrap resampling iterations, each drawing 80% of samples without replacement. K-means clustering (k = 3) was applied to each resampled dataset, and a consensus matrix was constructed by computing, for each pair of samples, the proportion of resampling iterations in which they were assigned to the same cluster. Cluster stability was quantified using the mean Jaccard similarity coefficient and the adjusted Rand index (ARI). The three-cluster solution was highly stable, with per-cluster Jaccard coefficients of 0.971, 0.989, and 0.965 (mean Jaccard = 0.975) and an ARI of 0.951 between the original and consensus cluster assignments. The consensus matrix exhibited a clear block-diagonal structure with high within-cluster co-assignment probabilities and low between-cluster probabilities (Fig. 6), indicating that the three metabolic phenotypes are reproducible and not driven by random initialization or dataset-specific noise.

### Association between sampling day and cluster membership

To assess whether the broad sampling window (− 7 to + 7 d relative to calving) introduced systematic bias in cluster assignment, a multinomial logistic regression was fitted with cluster membership as the outcome, sampling day as the predictor, and farm and parity as covariates (Cluster 3 as reference).

**Fig. 7:**
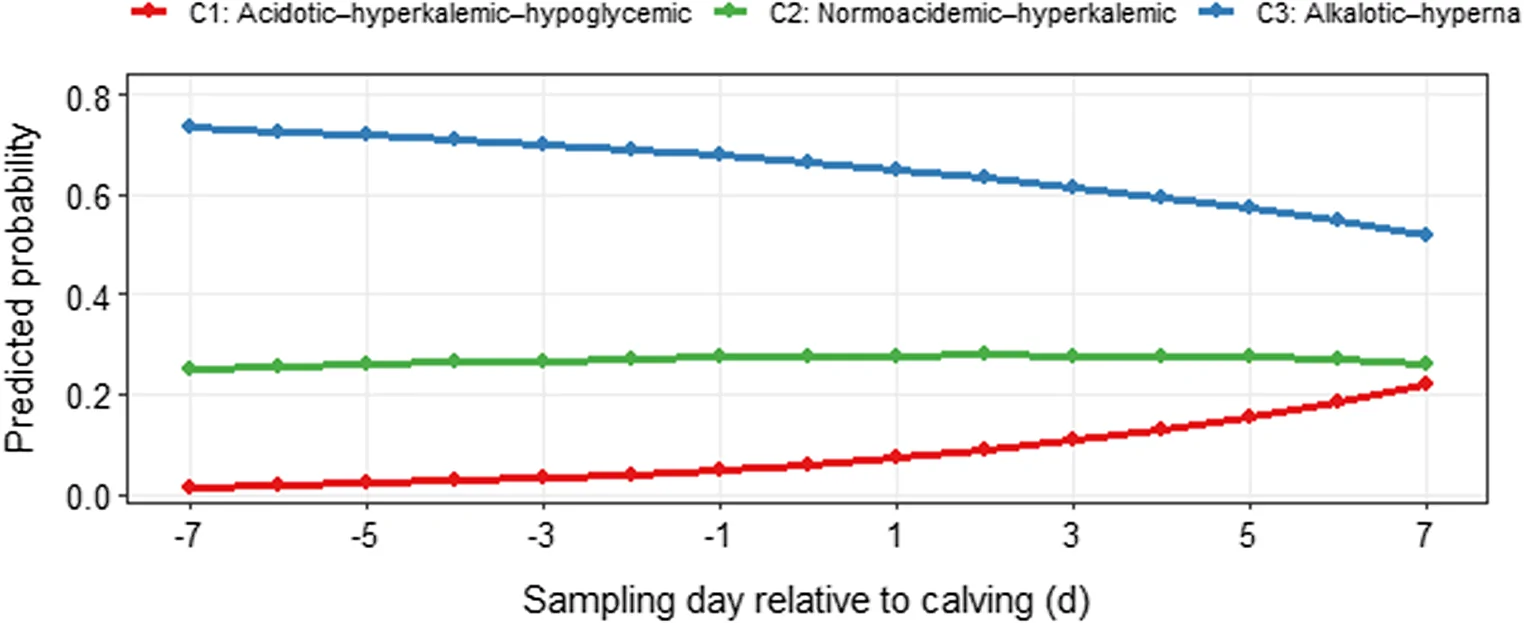
Predicted probabilities of metabolic cluster membership across the periparturient sampling window. Curves represent model-based predicted probabilities derived from a multinomial logistic regression with sampling day (days relative to calving) as the predictor and farm and parity as covariates. C1, Cluster 1; C2, Cluster 2; C3, Cluster 3. Cluster 3 served as the reference category in the regression model. The predicted probability of belonging to Cluster 1 increased progressively from antepartum to postpartum, whereas Cluster 2 remained relatively stable and Cluster 3 declined. Shaded areas are not shown; model-based confidence intervals are reported in the text

Sampling day was significantly associated with the likelihood of being classified into Cluster 1 (RRR = 1.18, 95% CI: 1.09–1.28, *P* < 0.001) but not Cluster 2 (RRR = 1.02, 95% CI: 0.96–1.09, *P* = 0.52). The likelihood ratio test comparing the full model with the reduced model without sampling day was significant (*χ²* = 16.84, df = 2, *P* < 0.001), indicating that sampling day contributed to cluster membership. These results are illustrated in Fig. 7, which shows the model-based predicted probabilities of cluster membership across the periparturient window. The predicted probability of belonging to Cluster 1 rose from approximately 5% at − 7 d to approximately 23% at + 7 d, whereas Cluster 2 remained stable at approximately 27–28% and Cluster 3 declined from approximately 74% to 53%.

However, the effect size was modest: each additional day postpartum increased the relative risk of belonging to Cluster 1 by approximately 18%, which translates to a cumulative increase of roughly 2.3-fold from − 7 d to + 7 d. Given that Cluster 1 prevalence ranged from 4.4% antepartum to 23.5% mid-postpartum (a 5.3-fold difference), sampling day explains only part of the observed variation.

Parity was a strong predictor: multiparous cows had 3.42 times the risk of being classified into Cluster 1 compared with primiparous cows (RRR = 3.42, 95% CI: 1.87–6.25, *P* < 0.001), consistent with the known greater susceptibility of multiparous cows to periparturient metabolic disorders. Farm ID also contributed significantly (*χ²* = 47.23, df = 12, *P* < 0.001), reflecting between-herd variation in management and nutrition.

When sampling day was included as a covariate in sensitivity analyses, the three-cluster structure remained qualitatively unchanged (data not shown), suggesting that while periparturient stage influences phenotype prevalence, it does not fully explain the separation among clusters.

### Metabolic heterogeneity in periparturient dairy cows revealed by unsupervised clustering

Based on the clustering results and statistical validation, the three distinct metabolic phenotypes were characterized by significant differences in key blood metabolites. The cluster centroids and inter-group comparisons are summarized below.

**Fig. 8:**
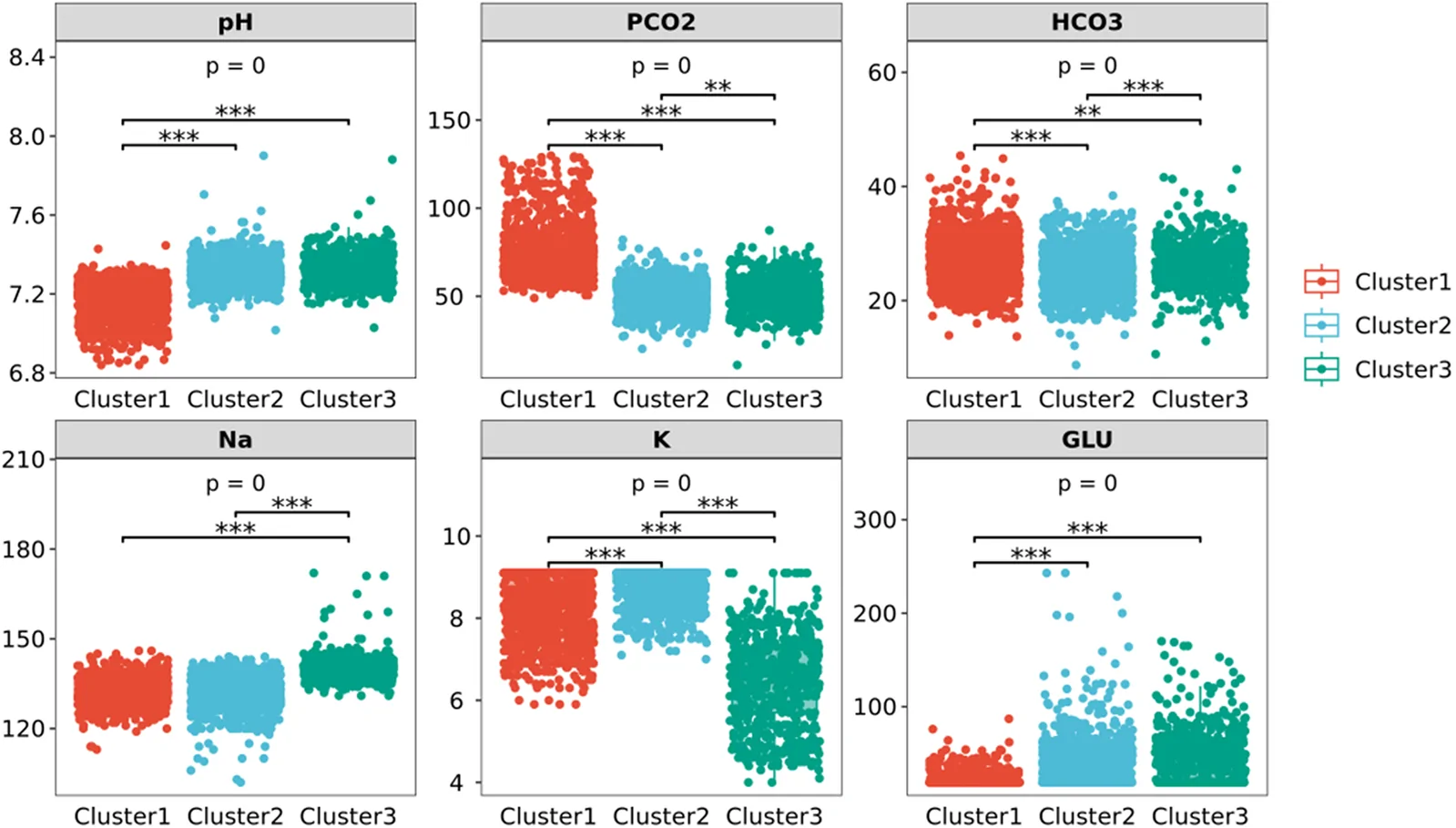
Distinct Metabolic Profiles Across Three Clusters in Periparturient Dairy Cows. Box-and-whisker plots showing the distribution of six key metabolic indicators (pH, PCO₂, HCO₃⁻, Na⁺, K⁺, and GLU) across three metabolically distinct clusters. Significant differences between clusters were assessed using one-way ANOVA followed by Tukey’s post-hoc test (*P* < 0.001, ***P < 0.001). Data points represent individual cows (n = 3,731 observations from 316 cows)

The mean values of six core metabolic indicators across the three clusters are presented in Fig. 8. Cluster 1 was defined by significantly lower pH (7.18), markedly elevated PCO₂ (74.53 mmHg), and higher K⁺ (8.60 mmol/L), along with the lowest GLU concentration (20.54 mg/dL). In contrast, Cluster 2 exhibited intermediate pH (7.297) and PCO₂ (51.22 mmHg), but maintained persistently high K⁺ (8.89 mmol/L) and moderate glucose levels (40.20 mg/dL). Cluster 3 demonstrated the highest pH (7.341), normal PCO₂ (51.33 mmHg), and significantly elevated Na⁺ (139.76 mmol/L) and GLU (51.32 mg/dL), with K⁺ within the reference range (6.78 mmol/L).

One-way ANOVA revealed statistically significant differences among the three clusters for all six variables (*P* < 0.001). Post-hoc Tukey HSD tests confirmed that Cluster 1 had significantly lower pH and higher PCO₂ and K⁺ compared to both Cluster 2 and Cluster 3 (*P* < 0.001). Cluster 3 exhibited significantly higher Na⁺ and GLU than the other two clusters (*P* < 0.001), while Cluster 2 showed no significant difference in pH or PCO₂ from Cluster 3 but remained distinct in electrolyte profile.

Biologically, these findings suggest that Cluster 1 represents a metabolically compromised metabolic state characterized by respiratory acidosis (evidenced by low pH and high PCO₂) combined with hyperkalemia and hypoglycemia, likely reflecting impaired ventilation, energy deficit, and metabolic stress during the periparturient period. Cluster 2 reflects a normoacidemic phenotype with isolated hyperkalemia, possibly indicating renal or hormonal dysregulation without overt acid-base imbalance. Cluster 3 demonstrates an alkalotic–hypernatremic–hyperglycemic profile, which may represent a compensated state driven by stress-induced gluconeogenesis and sodium retention, potentially associated with increased cortisol activity.

Based on the cluster centroid values and validated by exploratory ROC analysis, the metabolically compromised profile (Cluster 1) was associated with the following prepartum thresholds: venous pH < 7.25, PCO₂ > 58.75 mmHg, and glucose < 27.5 mg/dL. Potassium was not retained as a discriminatory threshold because its AUC was only 0.577. These thresholds should be considered exploratory and require validation in independent cohorts. Cows exhibiting two or more of these criteria during the late dry period (− 7 to − 1 d) should be considered at elevated risk for periparturient metabolic disorders.

### Dynamic shifts in the distribution of metabolic phenotypes across periparturient stages

**Fig. 9:**
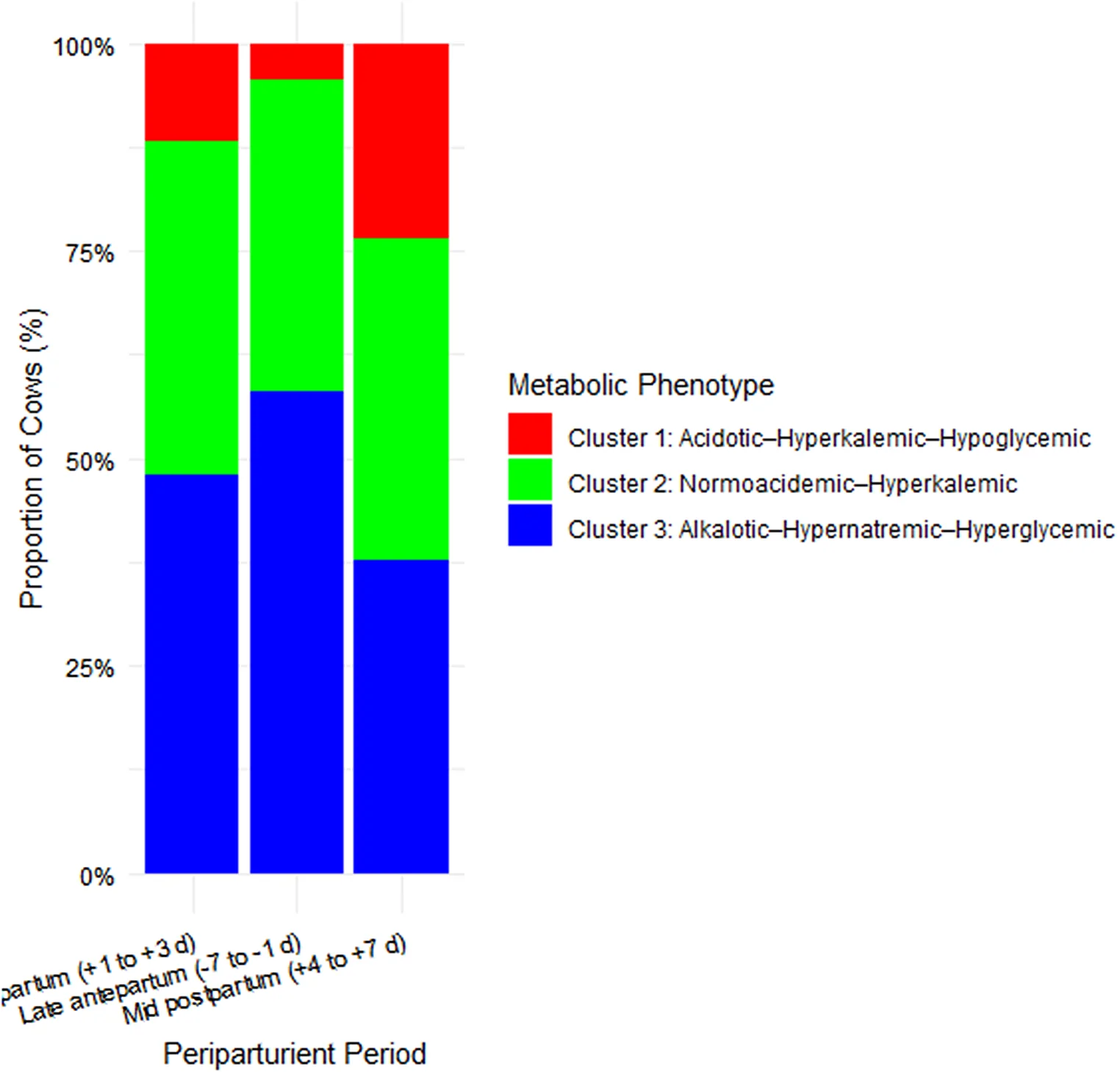
Distribution of metabolic phenotypes across periparturient stages. The proportion of cows classified into three distinct metabolic phenotypes during late antepartum (− 7 to − 1 d), early postpartum (+ 1 to + 3 d), and mid-postpartum (+ 4 to + 7 d) periods. Phenotypes were defined by k-means clustering based on blood parameters including pH, PCO₂, HCO₃⁻, Na⁺, K⁺, and glucose. Data are presented as the percentage of cows within each time window (*n* = 2,724 observations with complete data for farm and parity)

The distribution of metabolic phenotypes shifted dynamically across the periparturient period (Fig. 9). Cluster 3 predominated during late antepartum (57.9%), but its proportion declined postpartum (48.0% in early, 37.6% in mid postpartum). In contrast, the metabolically compromised Cluster 1 increased progressively from 4.4% antepartum to 23.5% by mid-postpartum. Cluster 2 remained consistently prevalent (ཞ38–40%) throughout all stages, suggesting persistent potassium dysregulation independent of acute acid-base status.

### Association of metabolic clusters with postpartum blood BHBA levels

**Fig. 10:**
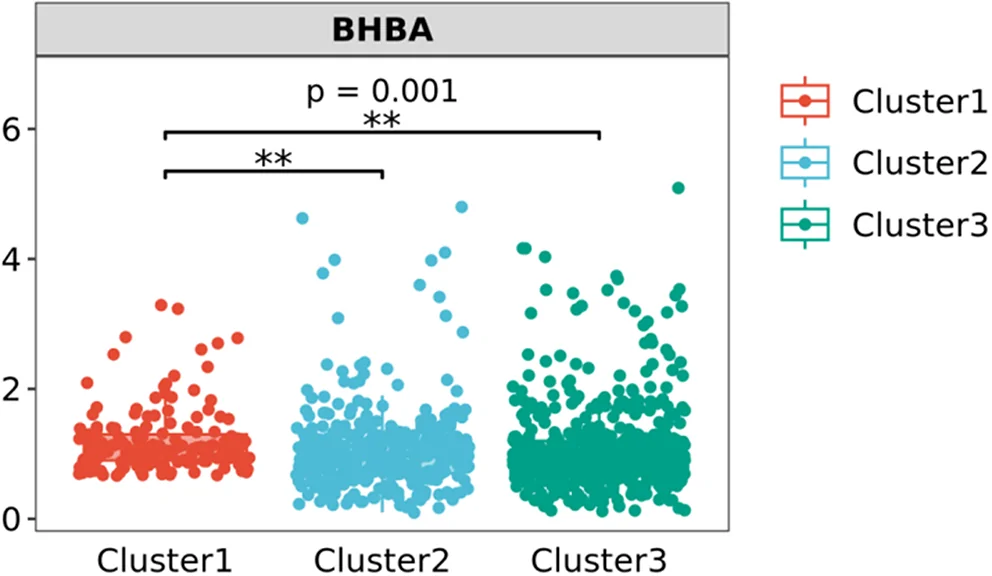
Blood β-hydroxybutyrate (BHBA) concentrations in periparturient dairy cows stratified by metabolic cluster (unadjusted analysis). Cows were assigned to Cluster 1, Cluster 2, or Cluster 3 based on unsupervised clustering of prepartum blood gas and biochemical parameters (*n* = 903 observations from 6 farms). Boxes represent interquartile ranges (25th–75th percentiles), horizontal lines indicate medians, whiskers extend to 1.5 × IQR, and dots denote outliers. After adjusting for farm in a linear mixed-effects model, the effect of cluster on BHBA was no longer significant (F = 0.48, *P* = 0.622)

Blood β-hydroxybutyrate (BHBA) concentrations were first compared across clusters using the Kruskal–Wallis test with Dunn’s post hoc correction (Fig. 10). In the unadjusted analysis, cows in Cluster 1 had modestly higher BHBA concentrations than those in Cluster 2 and Cluster 3 (median: 1.4 vs. 1.0 vs. 0.9 mmol/L; *P* = 0.001).

To account for potential herd-level confounding, we refitted the analysis using a linear mixed-effects model with farm as a random effect. BHBA data were available from 6 of the 7 farms (Farm1–Farm3 and Farm5–Farm7; *n* = 903). After adjusting for farm, the effect of cluster on BHBA was no longer significant (F = 0.48, *P* = 0.622). A sensitivity analysis using log-transformed BHBA yielded consistent results (F = 0.31, *P* = 0.730). Inspection of cluster-wise BHBA means across farms revealed that the apparent association in the unadjusted analysis was driven primarily by a small number of cows in Cluster 1 from a single farm (Farm1), whereas in the remaining five farms Cluster 1 did not show elevated BHBA relative to other clusters. These findings indicate that the apparent association between cluster membership and BHBA was largely attributable to herd-level variation, and that BHBA does not reliably discriminate among the three metabolic phenotypes.

### Milk composition and yield across metabolic clusters in early lactation dairy cows

**Fig. 11:**
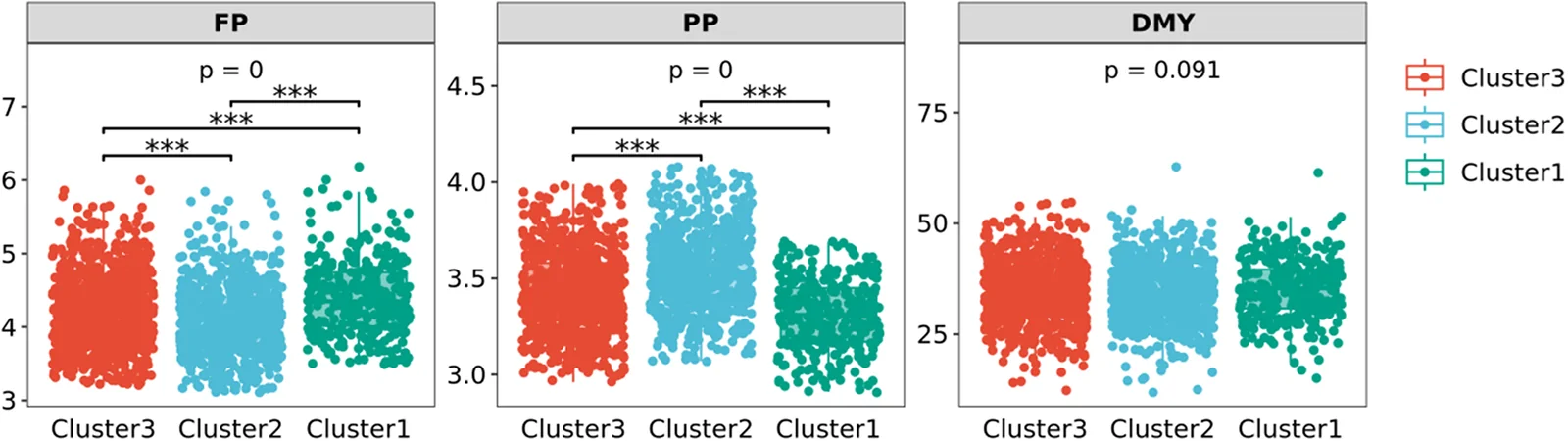
Milk composition and daily milk yield by metabolic cluster in early lactation dairy cows. Cows were classified into three metabolic phenotypes based on unsupervised clustering of prepartum blood gas and biochemical parameters: Cluster 1, Cluster 2, and Cluster 3. Milk fat percentage, milk protein percentage, and daily milk yield were recorded within 7 days postpartum. Boxes represent interquartile ranges (25th–75th percentiles), horizontal lines indicate medians, whiskers extend to the most extreme data points within 1.5 × interquartile range, and individual points denote outliers. Different letters above boxes indicate significant differences among clusters (*P* < 0.05, Kruskal–Wallis test with Dunn’s post hoc correction)

Milk composition varied significantly among metabolic clusters (*P* < 0.001). Cows in Cluster 3 had the highest milk fat percentage (5.8 ± 0.9%), followed by Cluster 2 (5.2 ± 0.8%) and Cluster 1 (4.6 ± 0.7%) (Fig. 11). A similar gradient was observed for milk protein percentage, which was greatest in Cluster 3 (3.9 ± 0.3%), intermediate in Cluster 2 (3.7 ± 0.3%), and lowest in Cluster 1 (3.3 ± 0.4%; *P* < 0.001). Despite these differences in composition, daily milk yield did not differ significantly across clusters (*P* = 0.091), although cows in Cluster 2 tended to produce less milk (32.1 ± 6.5 kg) than those in Cluster 1 (34.2 ± 7.1 kg) and Cluster 3 (34.8 ± 6.9 kg). Notably, the fat-to-protein ratio showed no significant variation among clusters (*P* = 0.21), indicating that reduced milk protein synthesis—rather than altered fat secretion—is the primary compositional consequence of the severe metabolic derangements characteristic of Cluster 1.

### Exploratory ROC analysis of candidate thresholds

**Fig. 12:**
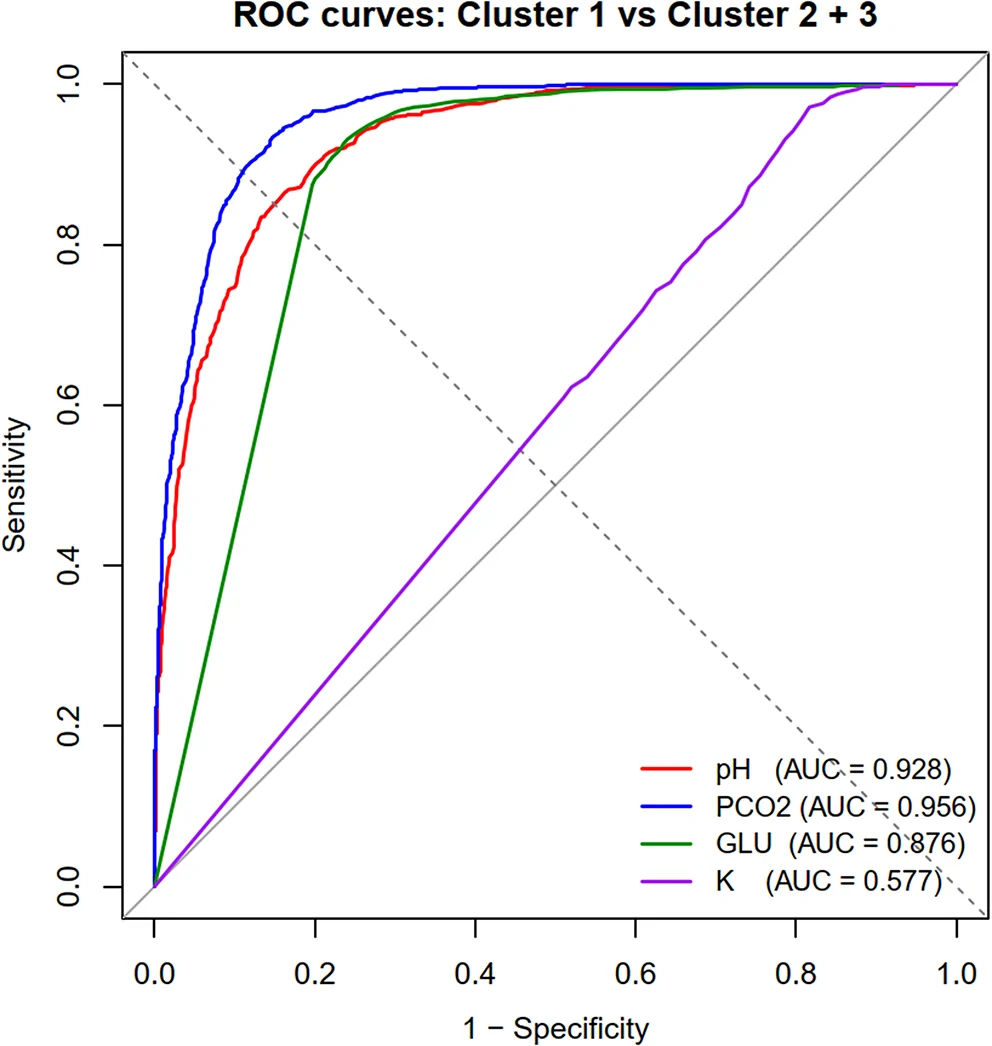
ROC curves for candidate prepartum thresholds. Receiver operating characteristic (ROC) curves for pH (red), PCO₂ (blue), GLU (green), and K⁺ (purple), using Cluster 1 vs. Cluster 2 + Cluster 3 as the binary outcome. The dashed diagonal line represents the reference line (AUC = 0.5). AUC values: pH = 0.928 (95% CI: 0.920–0.936); PCO₂ = 0.956 (0.950–0.962); GLU = 0.876 (0.866–0.886); K⁺ = 0.577 (0.561–0.593). pH, PCO₂, and GLU showed good to excellent discrimination, whereas K⁺ showed poor discrimination

To evaluate whether the cluster-derived thresholds could discriminate Cluster 1 from the other clusters, we performed receiver operating characteristic (ROC) analyses using Cluster 1 vs. Cluster 2 + Cluster 3 as the binary outcome. The AUC values were 0.928 for pH (95% CI: 0.920–0.936), 0.956 for PCO₂ (95% CI: 0.950–0.962), 0.876 for GLU (95% CI: 0.866–0.886), and 0.577 for K⁺ (95% CI: 0.561–0.593). The Youden-optimal thresholds were pH 7.255 (sensitivity 0.865, specificity 0.838), PCO₂ 58.75 mmHg (sensitivity 0.934, specificity 0.853), GLU 27.5 mg/dL (sensitivity 0.932, specificity 0.759), and K⁺ 6.85 mmol/L (sensitivity 0.971, specificity 0.183) (Fig. 12). pH, PCO₂, and GLU showed good to excellent discriminatory performance, whereas K⁺ showed poor discrimination (AUC = 0.577), with a sensitivity-specificity trade-off that was not clinically meaningful. A multivariable logistic model combining the four variables was attempted but exhibited quasi-complete separation, precluding stable maximum-likelihood estimation; therefore, only univariable ROC results are reported. These thresholds should be considered exploratory and require validation in independent cohorts before clinical application.

### Internal validation, supervised classification, and sensitivity analyses

Internal clustering quality was assessed using four complementary indices computed on the Euclidean distance matrix of the six standardized variables. The three-cluster solution yielded a Calinski–Harabasz index of 1,087.44, a Davies–Bouldin index of 1.448, a Dunn index of 0.010, and an average silhouette width of 0.231. The Calinski–Harabasz and Davies–Bouldin indices supported good cluster separation. The Dunn index was low, which is not unexpected in a high-dimensional dataset (six variables, *n* = 3,731 observations) where the maximum within-cluster diameter is sensitive to extreme values. The average silhouette width of 0.231 indicates weak-to-moderate cluster structure, consistent with the presence of some boundary samples between clusters, but the overall separation was supported by the highly stable consensus clustering results (mean Jaccard = 0.975; ARI = 0.951).

To evaluate whether cluster membership could be predicted from the six biomarkers, a random forest classifier (1,000 trees) was trained using cluster labels as the outcome. The classifier achieved an out-of-bag (OOB) accuracy of 98.1%, with class-specific error rates of 1.61% for Cluster 1, 1.66% for Cluster 2, and 3.45% for Cluster 3. Variable importance analysis identified K⁺ (MeanDecreaseAccuracy = 260.9), GLU (176.4), and PCO₂ (120.7) as the most informative predictors, whereas HCO₃⁻ showed the lowest importance (62.5), consistent with its partial redundancy with pH and PCO₂.

Leave-one-variable-out sensitivity analysis revealed that the three-cluster structure was partially sensitive to feature removal. Removing Na⁺ (ARI = 0.897) or HCO₃⁻ (ARI = 0.868) had minimal effect on cluster assignments, whereas removing pH (ARI = 0.482), GLU (ARI = 0.593), or PCO₂ (ARI = 0.619) led to moderate changes. Removing K⁺ had the largest effect (ARI = 0.329), indicating that K⁺ is the single most influential variable in maintaining the three-cluster structure, consistent with its high importance in the random forest classifier. These results indicate that the clustering solution depends primarily on K⁺ and pH, while Na⁺ and HCO₃⁻ are largely redundant with other variables.

## Discussion

In this study, unsupervised clustering of integrated blood gas and electrolyte profiles from a multi-farm cohort of periparturient dairy cows identified three distinct metabolic phenotypes. Cluster 1 represented a metabolically compromised state, characterized by low pH, elevated PCO₂, reduced HCO₃⁻, hypoglycaemia, and electrolyte imbalance—consistent with respiratory acidosis coupled with impaired energy metabolism. Cluster 2 exhibited a normoacidemic but hyperkalemic pattern, with intermediate pH and PCO₂ and persistently elevated potassium, suggesting isolated electrolyte dysregulation without overt acid–base disturbance. Cluster 3 displayed a homeostatic profile, characterized by alkalotic tendencies, elevated sodium and glucose levels, and stable acid–base balance. Notably, a substantial proportion of cows assigned to the compromised phenotype (Cluster 1) were sampled during the antepartum period, indicating that significant metabolic dysregulation can be detected before calving, well in advance of clinical disease onset.

The identification of quantitative thresholds for cluster 1 provides a practical framework for on-farm risk stratification. Unlike single-biomarker approaches, this multi-parameter definition captures the synergistic effects of acidosis, hypoglycemia, and electrolyte imbalance—pathophysiological hallmarks that precede clinical ketosis, displaced abomasum, or metritis. Portable blood gas analyzers enable rapid assessment of these parameters within minutes. However, whether targeting this profile with interventions such as propylene glycol supplementation or dietary cation–anion balance adjustment improves clinical outcomes remains to be determined in future intervention studies.

Our identification of a prepartum acidotic and hypoglycaemic phenotype aligns with and extends previous observations linking acid–base disturbances to transition cow health. Subclinical acidosis during late gestation is associated with an increased risk of displaced abomasum and ketosis postpartum (Rodríguez et al. 2017, Vallejo-Timarán et al. 2020). Disruptions in the acid–base balance, as reflected by low pH and high PCO_2_, appear to be closely associated with the development of hepatic steatosis and insulin resistance in periparturient cows (Sadri and Lautt 1999). Our findings further suggest that these abnormalities are not isolated deviations but coalesce into a coherent identifiable physiological state that can be objectively stratified using routine on-farm diagnostics.

The presence of hypoglycaemia in cluster 1 was particularly noteworthy. Although GLU is traditionally considered as a downstream marker of NEB, its concurrent decline with acidosis suggests profound metabolic inflexibility. Under severe NEB, excessive lipolysis leads to elevated NEFA flux to the liver, promoting ketogenesis and potentially impairing gluconeogenic capacity (Wen et al. 2025). Acidosis also suppresses hepatic GLU production and reduces peripheral GLU utilisation, creating a vicious cycle that exacerbates energy deficit (Imenez Silva and Mohebbi 2022). The observed hyperkalaemia in cluster 1 may reflect cellular stress or insulin deficiency, as insulin normally promotes K⁺ uptake into cells; its reduction during NEB possibly contributes to elevated serum K⁺ levels (Li and Vijayan 2014, Yamada and Inaba 2021).

The co-occurrence of acidosis, hypoglycemia, and hyperkalemia in cluster 1 reflects a systemic failure of metabolic homeostasis with direct pathophysiological consequences. Acidosis impairs hepatic gluconeogenesis and reduces peripheral glucose utilization, exacerbating energy deficit and promoting lipolysis. Concurrently, insulin deficiency—common under severe negative energy balance—diminishes cellular potassium uptake, contributing to hyperkalemia. This triad creates a vicious cycle: low glucose further suppresses insulin secretion, while acidosis worsens electrolyte dysregulation. Clinically, this state predisposes cows to ketosis (via uncontrolled ketogenesis), displaced abomasum (due to smooth muscle dysfunction from acidosis and hypokalemia paradox), and immunosuppression (from energy deprivation in immune cells). It should be noted that although the unadjusted analysis showed higher BHBA concentrations in cluster 1, this association was not robust to adjustment for farm of origin (F = 0.48, *P* = 0.622), and the apparent association was driven primarily by a small number of cluster 1 cows from a single farm. Therefore, BHBA should not be considered a reliable discriminator of the three metabolic phenotypes in this cohort. Critically, our data show that this metabolically compromised phenotype is identifiable up to 7 days before calving, offering a window for preemptive management.

In contrast, the normoacidemic–hyperkalemic profile (cluster 2) may arise from distinct mechanisms, such as aldosterone insufficiency or renal tubular dysfunction, which do not directly impair energy metabolism but may still affect lactational performance through reduced feed intake or altered fluid balance. Although the milk fat-to-protein ratio is widely used as a proxy for negative energy balance, its utility may be limited in herds with uniformly high milk fat content or impaired hepatic function. In our cohort, reduced milk protein percentage emerged as the more discriminative indicator of prepartum metabolic stress, aligning with the hypoglycaemic state of cluster 1 cows that limits amino acid uptake by the mammary gland. These mechanisms were not directly measured in this study and should be considered speculative.

Importantly, approximately 30% of antepartum samples already belonged to the compromised phenotype in this study, challenging the conventional view that metabolic crises emerge only after calving. This implies that the roots of peripartum disorders lie in late gestation, possibly driven by factors such as overconditioning, inadequate management of dietary cation–anion differences, or chronic subacute ruminal acidosis. Therefore, early identification of at-risk cows before parturition is important for implementing targeted interventions, such as adjusting prepartum diets or improving monitoring protocols. Future studies, including intervention trials, are needed to determine whether these strategies improve clinical outcomes (Zwierzchowski et al. 2024).

This study has some limitations. First, our analysis was based on a single blood draw per cow, providing only a snapshot of the dynamic periparturient physiology. Therefore, longitudinal profiling should be performed to better characterise phenotypic stability and transitions. Second, although we controlled for parity and farm in descriptive analyses, we did not include them as covariates in clustering because of the unsupervised nature of the approach; future supervised models should integrate these factors. Third, clinical outcomes were not tracked in this cohort, limiting the direct validation of the predictive value of cluster 1. Future studies should link these metabolic phenotypes to specific health and production endpoints.

Several limitations related to variable selection should be acknowledged. First, the clustering framework was restricted to six variables measurable by a point-of-care blood gas analyser, and did not include NEFA, chloride, lactate, or magnesium. NEFA in particular is a cornerstone biomarker of negative energy balance, and its inclusion might have refined the characterisation of the “metabolically compromised” phenotype or revealed additional sub-phenotypes not captured by the current panel. Chloride, lactate, and magnesium could likewise have contributed to a more complete assessment of strong ion balance, ruminal acidosis, and neuromuscular metabolic regulation, respectively. Second, ionised calcium was measured but excluded from the final feature set due to collinearity with acid–base parameters; consequently, the present phenotypes do not explicitly capture subclinical hypocalcaemia, which is highly prevalent in multiparous transition cows and may interact with the acidotic phenotype identified here. Third, the six-variable solution reflects a deliberate trade-off between physiological comprehensiveness and field practicality: it enables immediate on-farm application but necessarily omits biomarkers that require laboratory-based assays. Future studies incorporating NEFA, chloride, lactate, magnesium, and ionised calcium—ideally in combination with the six variables retained here—are warranted to validate and extend the present phenotypes, and to determine whether a broader biomarker panel improves prepartum risk discrimination beyond the thresholds proposed in this study.

The cross-sectional design represents a further limitation. Because each cow was sampled only once during the periparturient window, the study cannot characterise within-individual temporal changes in metabolic phenotype, nor can it determine whether individual cows transition between the three identified clusters as calving approaches or as lactation progresses. The observed shift in phenotype distribution from antepartum to mid-postpartum (Fig. 9) therefore describes population-level patterns across independently sampled cows rather than trajectories within the same animals. This design also precludes assessment of the temporal sequence between the prepartum acidotic profile and postpartum outcomes such as elevated BHBA or reduced milk protein, which would require repeated sampling of the same individuals. Future longitudinal studies with serial blood gas and electrolyte measurements from late dry-off through early lactation are needed to confirm whether the prepartum metabolically compromised profile identified here represents a stable individual trait, a transient state, or a progressive deterioration, and to establish the optimal sampling window for early intervention.

Regarding variable selection, multicollinearity was formally assessed using VIF and condition indices in addition to Pearson correlations. While this approach identified and excluded severely collinear variables, it should be noted that correlation-based and VIF-based criteria are not equivalent to causal or biological variable selection. Some variables excluded on statistical grounds may carry independent physiological information that could refine phenotype characterisation in future studies with larger sample sizes or alternative modelling approaches such as regularised regression or partial least squares. Nevertheless, the six retained variables showed acceptable collinearity diagnostics (all VIF < 2.5; maximum condition index = 8.2), supporting the stability of the clustering solution.

Regarding data preprocessing, the sequential order of outlier removal and Z-score standardisation was explicitly defined to ensure reproducibility. Outliers were removed on the raw scale before standardisation, which prevents extreme values from distorting the mean and standard deviation used for Z-score calculation. However, this order also means that the final standardised dataset is conditional on the outlier removal step; if different outlier criteria had been applied, the standardised values and potentially the clustering solution could differ. Sensitivity analyses using alternative outlier thresholds (1.5 × IQR and 2 × IQR) yielded comparable three-cluster solutions, supporting the robustness of the present approach. Future studies should report the full preprocessing pipeline and consider sensitivity analyses across different preprocessing choices.

Leave-one-variable-out sensitivity analysis showed that the clustering solution was partially sensitive to feature removal, with K⁺ and pH having the largest effects (ARI = 0.329 and 0.482, respectively, when removed). This suggests that the three-cluster structure depends primarily on these two variables and that future studies with broader biomarker panels should examine whether the phenotypes remain stable when additional variables are included.

## Conclusions

Periparturient dairy cows segregated into three candidate metabolic profiles based on integrated blood gas and electrolyte parameters. The metabolically compromised profile (Cluster 1) was characterized by prepartum acidosis, hypoglycemia, and hyperkalemia and was identifiable up to one week before calving. This profile was associated with reduced milk protein percentage, whereas its association with elevated postpartum β-hydroxybutyrate was not robust to adjustment for herd-level effects, suggesting that reduced milk protein is a more consistent indicator of impaired metabolic adaptation in this phenotype. Based on cluster centroids and exploratory ROC analysis, the following prepartum thresholds were proposed for identifying the metabolically compromised profile: venous pH < 7.25 (AUC = 0.928), PCO₂ > 58.75 mmHg (AUC = 0.956), and glucose < 27.5 mg/dL (AUC = 0.876). Potassium was not retained as a discriminatory threshold because its AUC was only 0.577. These thresholds are exploratory and require validation in independent cohorts before they can be implemented as diagnostic or management tools. Future intervention studies are also needed to determine whether targeting this profile improves clinical outcomes in transition dairy cows.

## Data Availability

The data presented in this study are available on request from the corresponding author. The raw sequencing and phenotypic datasets are not publicly archived due to ongoing analyses and proprietary agreements with participating farms; however, de-identified metabolic and clustering results can be shared upon reasonable request.
